# The Role of Micronutrients in Support of the Immune Response against Viral Infections

**DOI:** 10.3390/nu12103198

**Published:** 2020-10-20

**Authors:** Francesco Pecora, Federica Persico, Alberto Argentiero, Cosimo Neglia, Susanna Esposito

**Affiliations:** Pediatric Clinic, Pietro Barilla Children’s Hospital, Department of Medicine and Surgery, University of Parma, via Gramsci 14, 43126 Parma, Italy; cescopec@hotmail.it (F.P.); federica.persico@studenti.unipr.it (F.P.); aargentiero85@gmail.com (A.A.); negliamino@gmail.com (C.N.)

**Keywords:** micronutrients, immune system, respiratory viral infections, vitamins, minerals

## Abstract

Viral infections are a leading cause of morbidity and mortality worldwide, and the importance of public health practices including handwashing and vaccinations in reducing their spread is well established. Furthermore, it is well known that proper nutrition can help support optimal immune function, reducing the impact of infections. Several vitamins and trace elements play an important role in supporting the cells of the immune system, thus increasing the resistance to infections. Other nutrients, such as omega-3 fatty acids, help sustain optimal function of the immune system. The main aim of this manuscript is to discuss of the potential role of micronutrients supplementation in supporting immunity, particularly against respiratory virus infections. Literature analysis showed that in vitro and observational studies, and clinical trials, highlight the important role of vitamins A, C, and D, omega-3 fatty acids, and zinc in modulating the immune response. Supplementation with vitamins, omega 3 fatty acids and zinc appears to be a safe and low-cost way to support optimal function of the immune system, with the potential to reduce the risk and consequences of infection, including viral respiratory infections. Supplementation should be in addition to a healthy diet and fall within recommended upper safety limits set by scientific expert bodies. Therefore, implementing an optimal nutrition, with micronutrients and omega-3 fatty acids supplementation, might be a cost-effective, underestimated strategy to help reduce the burden of infectious diseases worldwide, including coronavirus disease 2019 (COVID-19).

## 1. Introduction

The immune system defends the body against infectious agents and other internal and external insults. The immune defense system comprises a combination of anatomic physical barriers, including the skin, mucous membranes, mucous blanket, and ciliated epithelial cells [1]. If these are evaded, the components of the immune system are quickly activated to protect the body against any “non-self” molecules. The immune system integrates two fundamental response mechanisms: the innate response and the acquired one. Innate responses occur to the same extent however many times the infectious agent is encountered. Innate immunity is rapid and utilizes receptors (pattern recognition receptors, PRRs) to recognize the invading particles, known as pathogen-associated molecular patterns (PAMPs). The innate defenses comprise cell-intrinsic responses to viral infections, leukocyte responses to pathogens, and soluble mediators such as complement proteins. Innate immunity is an immediate defense that is not as specific or mutable as antigen receptors. Adaptive immunity is specific to T and B cells. These cells recognize specific antigens on the invading microorganism and produce antibodies to target and destroy the pathogen, enabling identification for attack by other immune cells or neutralizing the pathogen directly. The adaptive immunity generates the immunological memory; thus, acquired responses improve on repeated exposure to a given infection [2,3,4].

It is today acknowledged that an adequate nutritional status is crucial for the development, maintenance, and expression of the immune response [5,6]. Micronutrients (i.e., vitamins and nutritionally essential minerals) influence and support every stage of the immune response. Deficiencies of micronutrients can affect both innate and adaptive immunity, causing immunosuppression and thus increasing the susceptibility to infections. In addition, mucosal-associated invariant T cells (MAIT), which are innate-like T cells expressing a semi-invariant T cell receptor, play a role in polarizing adaptive lymphocyte function, and contribute to metabolic dysfunction [7]. Furthermore, infections and an inadequate nutritional status have a synergistic relationship. The immune response itself to an infection exacerbates a poor nutritional state and causes an increase in the demand for micronutrients [8,9]. Viral infections are a leading cause of morbidity and mortality worldwide [10], as is shown by both seasonal influenza, and the recent outbreak of coronavirus disease 2019 (COVID-19), caused by the novel severe acute respiratory syndrome coronavirus 2 (SARS-CoV-2) [11].

Many people of all ages have single or multiple micronutrient deficiencies. Supplementation of micronutrients may play an important role in enhancing the resistance to infections, restoring the immune function. The main objective of this manuscript is to discuss our present knowledge of the efficacy of micronutrients in supporting immunity, particularly with respect to respiratory virus infections. PubMed was used to search for all of the studies published up to April 2020 using keywords such as “Micronutrients” and “Immune system” and “Micronutrients” and “Respiratory viral infections”. The search was limited to articles published in English that provided evidence-based data.

## 2. The Role of Micronutrients against Virus Infection

Micronutrients, including several vitamins (vitamin A, B6, B12, folate, C, D, E) and trace elements (Zinc, Selenium, Copper, Magnesium), play important roles in supporting the immune system, and thus their deficiencies could increase the susceptibility of a host to infectious diseases [12]. Adequate levels of micronutrients are essential to ensure an effective function of each component of the immune system. Regarding the innate immunity, micronutrients play fundamental roles in maintaining the structural and functional integrity of the physical barriers, such as skin and mucus membranes. Micronutrients are also involved in supporting activity of antimicrobial proteins and chemotaxis of innate cells. Furthermore, several vitamins and minerals contribute to the phagocytic and killing activities of neutrophils and macrophages [13]. Deficiencies of vitamins and select essential minerals also affect several aspects of the adaptive immunity, in particular the humoral response (antibody-mediated) and the cell-mediated immunity.

It is acknowledged that an impaired nutritional status increases the susceptibility and the severity of infections. In turn, serious or repeated infections increase the risk of malnutrition, by inducing anorexia with associated decreased intake of nutrients, causing a status of malabsorption, or altering the body’s metabolism and increasing demand for nutrients [14]. Therefore, it is essential to maintain adequate amounts of each micronutrient. A well-balanced diet is crucial to achieve an optimal intake of all these vitamins and essential elements. However, in the general population and also in developed countries, it can be difficult to obtain an adequate micronutrient intake versus the Recommended Daily Allowance (RDA), because of reduced intake, increased requirements for metabolism, and increased loss.

**Figure 1:**
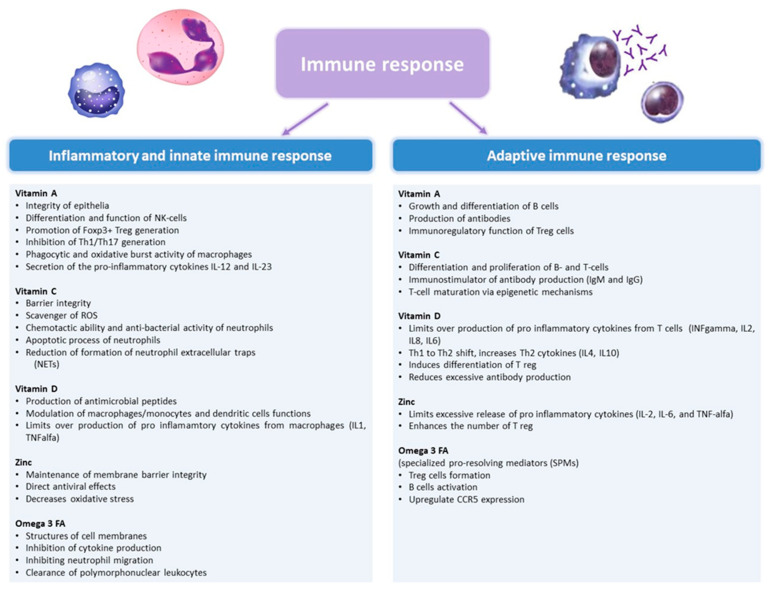
The role of vitamin A, C, D, Zinc, and Omega-3 fatty acids in the immune response. IFN, interferon; IL, interleukin; ROS, reactive oxygen species; TNF, tumor necrosis factor.

We reviewed some of the above-mentioned micronutrients that may play an important role in supporting immune functions (Figure 1), thus influencing risk and clinical course of viral respiratory infections.

## 3. Vitamin A

### 3.1. Metabolism and Functions

Retinol (vitamin A_1_) is a fat-soluble vitamin and an obligatory dietary factor since it is not synthesized de novo by humans. The main vitamin A sources are organ meats, milk, cheese; in green vegetables and yellow fruits are present provitamin A carotenoids, which must be cleaved to retinal before absorption [15]. Preformed vitamin A (retinol, retinal, retinoic acid, and retinyl ester) is hydrolysed into retinol in the lumen of the small intestine. Retinol is esterified in the enterocyte and packaged into chylomicrons, and the liver represents the main site of chylomicron vitamin A storage. During a deficiency status, vitamin A stores are mobilized, and retinol circulate bound to the retinol-binding protein (RBP) and is utilized by target tissues [15].

The functions of vitamin A are mediated by all-trans-retinoic acid, which, by binding specific nuclear transcription factors, (retinoid receptors) regulates the expression of several hundred genes [15,16,17,18,19]. Vitamin A-regulated genes are involved in fundamental biological activities, playing an important role in supporting vision, growth, cell, and tissue differentiation, haematopoiesis and immunity.

Regarding immunity, vitamin A contributes to supporting the integrity of epithelia, particularly the gastrointestinal epithelia tissue among children suffering from severe infections or who are undernourished [15]. Vitamin A is also important in regulating the number and function of natural killer (NK) cells, macrophages, and neutrophils [16,17]. By downregulating the expression level of interferon (IFN)-γ and upregulating the secretion of inerleukin (IL)-5, vitamin A plays a regulatory role in the early differentiation stage of NK cells. Moreover, it regulates the differentiation of dendritic cells precursors and promotes the secretion of the pro-inflammatory cytokines IL-12 and IL-23 by dendritic cells. It has also a crucial role in promoting Foxp3+ Treg generation, while reciprocally inhibiting Th1/Th17 generation and a Th9 transcriptional and epigenomic program [20,21]. Furthermore, vitamin A is involved in the antimicrobial action of macrophages, playing a role in the phagocytic and oxidative burst activity [13].

Vitamin A also supports adaptive immunity. Indeed, retinoids represents physiological modulators of normal B cell growth and differentiation, thus vitamin A deficiency negatively affects B cell function [18]. Furthermore, animal studies have shown impairment in the antibody response due to vitamin A deficiency [19]. The production of antibodies may be enhanced by the influence of vitamin A on T helper 2 cells development [22] and antigen-presenting cells [15,23]. In addition, retinoids induce the differentiation of Tregs and maintain both the stability of Tregs and their immunoregulatory function [24]. Indeed, retinoids play fundamental roles in cell-mediated immunity, representing an important cofactor in T cell activation [25] and influencing the expression of membrane receptors that mediate T-cell signalling [17]. Vitamin A supplementation trials conducted in paediatric populations have shown the potential effect to increase T-cell, particularly of the CD4 subpopulation [15,26].

### 3.2. Vitamin A Status

Vitamin A deficiency states in developed countries are rare, but many developing countries have vitamin A deficiency of public health significance, associated with overt signs of deficiency, or subclinical levels of vitamin A depletion with marginal liver reserves [27]. However, today, marginal vitamin A status is prevalent and difficult to diagnose. Vitamin A biomarkers have been developed to diagnose different degrees of vitamin A status [28]. There are biological and functional indicators, such as ophthalmic signs of vitamin A deficiency (night blindness, xerophtalmia, Bitot spots), and biochemical indicators. As biochemical indicators, serum retinol concentrations are the most common population indicator. Normal plasma levels are 20–50 µg/dL in infants and increase gradually as children become older. However, serum retinol concentration is affected by infection and inflammation because RBP is an acute phase protein, thus these conditions may mimic a lack of vitamin A. For this reason, RBP linked with markers of inflammation may be used to adjust serum retinol concentration even if the ratio of retinol to RBP is influenced by a status of vitamin A deficiency, because of the increased level of circulating unbound plasma RBP. Furthermore, serum retinol concentration is homeostatically controlled over a wide range of liver reserves, and thus does not reflect the vitamin A liver stores. However, in children, values <10 µg/dL indicate a deficiency of vitamin A [29].

### 3.3. Recommended Daily Allowance and Supplementation

The recommended daily allowance for vitamin A is 450 µg for infants up to 12 months of age. The dietary reference intakes in older children are different based on age-sex group (Table 1).

Compared with those in adults, neonates present low levels of vitamin A, and lower vitamin A stores and plasma retinol concentrations are seen in low-birthweight infants and in preterm newborns. Thus, vitamin A is used in preterm infants, due to the demonstrated efficacy in improving respiratory function and preventing development of chronic lung disease [30,31]. Furthermore, supplementation with vitamin A is recommended in case of latent vitamin A deficiency using a dose of 1500 µg daily. In children at risk of vitamin A deficiency, rates of mobility and mortality, probably associated with viral infection such as measles, have been reduced by a weekly doses of vitamin A at the RDA level [29,32].

### 3.4. Vitamin A Supplementation against Viral Infections

Vitamin A supplementation is correlated with a reducing in the infection-related morbidity and mortality associated with vitamin A deficiency (Table 2) [33,34,35,36,37,38,39,40,41,42,43].

A meta-analysis of 47 studies that included 1,233,856 children found that vitamin A supplementation is associated with a reduction of all-cause mortality of 12% [33]. This association could be explained by the low-to-moderate evidence that vitamin A supplementation in children can reduce the incidence of diarrhea and measles [34,35]. In contrast with previous several trials [33], in a large cluster-randomized trial (DEVTA trial) that included more than 1 million pre-school children in North India, a region with high frequency of vitamin A deficiency, supplementation with high-dose vitamin A (200,000 UI every 6 months] did not achieve a significant mortality reduction. However, in the same article, a meta-analysis that included DEVTA plus eight previous randomised trials of supplementation yielded a weighted average mortality reduction of 11% [32]. Moreover, although it has been reported that there is a significant association between low serum concentration of retinol and acute lower respiratory tract infections [36], several studies in children, particularly regarding the role of vitamin A for treatment of respiratory syncytial virus infection [37,38,39], have shown that vitamin A supplementation is not effective in reducing the incidence of lower respiratory tract infections [40,41,42,43]. However, considering the evidence of the role of vitamin A in supporting an effective immune system, and of the effect of vitamin A on child mortality, supplementation should be offered to children in population at risk of vitamin A deficiency, which could also include patients with disorders associated with fat malabsorption.

## 4. Vitamin C

### 4.1. Metabolism and Functions

Vitamin C (ascorbic acid) is a water-soluble vitamin and an essential micronutrient for humans. The main sources of vitamin C are citrus fruit, tomatoes, potatoes, and green leafy vegetables. The provision of dietary vitamin C is dependent on food preparation because it is easily destroyed by prolonged storage, overcooking, and processing of foods. Breast milk represents an adequate source of vitamin C for newborns and infants.

Ascorbic acid is an antioxidant (electron donor) and is involved in several biological processes: synthesis of collagen, neurotransmitter metabolism, cholesterol metabolism, fatty acid transport (synthesis of carnitine), maintaining the iron and copper atoms, and is a cofactor of the metalloenzymes, in a reduced active state. Furthermore, vitamin C affects the cellular and immunologic functions of the hematopoietic system, due to its role in enhancing nonheme iron absorption, the transfer of iron from transferrin to ferritin, and the formation of tetrahydrofolic acid [44].

Vitamin C is an essential nutrient that influences several aspects of the immune system, particularly barrier integrity and leukocyte function [45]. The fact that vitamin C is actively accumulated into the epidermal and dermal cells and into leukocytes, via sodium-dependent transporter, suggests that the vitamin plays a crucial role within the skin and the leukocytes [46]. Vitamin C is a potent water-soluble antioxidant and plays an important role in maintaining redox homeostasis within cells and in protecting host cells against the actions of reactive oxygen species (ROS) [45], released by phagocytes in order to lead to the deactivation of viruses and the killing of bacteria. Thus, acid ascorbic as scavenger of ROS may both protect crucial cell structural components and modulate the pro-inflammatory signaling pathway activated by the oxidative burst [46,47]. Vitamin C influences innate immunity also by regulating several aspects of neutrophil function [48], particularly the chemotactic ability, as shown in several in vitro and in vivo animal studies [49,50]. Severe septic syndromes are associated with impaired neutrophil chemotactic ability [51], and studies conducted in children and neonates may suggest that it could be due also by a severe infection-induced status of vitamin C deficiency [52,53]. Furthermore, studies have shown that, in patients with recurrent infections or affected by genetic conditions such as Chediak–Higashi syndrome (CHS), supplementation with vitamin C improved significantly the antibacterial activity of neutrophils [54,55]. Ascorbic acid may also influence the apoptotic process of neutrophils, thus promoting resolution of inflammation and reducing extensive tissue damage [46]. Lastly, promising in vitro and preclinical data suggest that vitamin C supplementation could play a role on more recently discovered functions of neutrophils, such as the formation of neutrophil extracellular traps (NETs), resulting by the release of toxic intracellular components following the necrotic death of neutrophils [56]. Ascorbic acid may attenuate tissue damage reducing the formation of NETs [48].

Furthermore, vitamin C is effective in supporting both the humoral response and the cell-mediated immunity [46]. Vitamin C accumulates in phagocytic cells, such as neutrophils, and can enhance chemotaxis, phagocytosis, generation of ROS, and ultimately microbial killing [57]. It is also needed for apoptosis and clearance of the spent neutrophils from sites of infection by macrophages, thereby decreasing necrosis/NETosis and potential tissue damage. The role of vitamin C in lymphocytes is less clear, but it has been shown to enhance differentiation and proliferation of B- and T-cells, likely due to its gene-regulating effects. The effect of vitamin C on cytokine generation appears to depend on the cell type and/or the inflammatory stimulant. Recent research has indicated that vitamin C treatment attenuates synthesis of the pro-inflammatory cytokines TNF, IL-6, and IL-1β [57]. In vitro studies suggest that ascorbic acid operates as potent immunostimulator of antibody production (IgM and IgG) in humans and that the intracellular ascorbic acid content is a key parameter for establishing the immune response of peripheral blood lymphocytes [58]. Other in vitro studies have shown the role of vitamin C in promoting T-cell maturation [59]. Recent research indicates the possible role of vitamin C in regulating T-cell maturation via epigenetic mechanisms involving in the ten-eleven translocations (TETs) and histone demethylation [60,61].

### 4.2. Vitamin C Status

Normal plasma vitamin C concentration is 50 µmol/L. Plasma ascorbate concentrations above 10 µmol/L but below 50 µmol/L represent a status with an increased risk of insufficiency. Scurvy appears when the plasma concentration falls below 10 µmol/L, which corresponds to an intake of less than 10 mg vitamin C/day and a body pool less than 300 mg [60]. It is acknowledged that vitamin C concentration declines during stress and infection, particularly in leukocytes, as it is used to protect host cells against the oxidative stress [46]. For the same reason, children exposed to smoking or environmental tobacco smoke require increased intake of vitamin C [44].

### 4.3. Recommended Daily Allowance and Supplementation

In adults, the average requirement of vitamin C is considered to be the amount that compensates for the metabolic losses of vitamin C and ensures a fasting ascorbate plasma level of 50 µmol/L [62,63] (Table 1).

In infants and children, no data for deriving the average requirement are available. The European Food Safety Authority (EFSA) extrapolated the vitamin C requirement in this age group from the vitamin C requirement in adult [62].

### 4.4. Vitamin C Supplementation against Viral Infection

Vitamin C deficiency status is correlated with an increased susceptibility to severe respiratory infections such as pneumonia [46,47,64,65,66,67,68,69,70,71,72] (Table 3).

In a recent meta-analysis, Hemilä and Louhiala analyzed the effect of vitamin C in preventing and treating pneumonia regardless of the etiology [65]. They reported three studies that show a >80% lower incidence of pneumonia in the vitamin C groups, supporting the potential role of vitamin C in reducing the risk of pneumonia, particularly in individuals with low plasma vitamin C levels [66]. Furthermore, regarding the effect of vitamin C in treating pneumonia, in older patients, lower mortality and reduced severity of disease was found in the vitamin C group, particularly in the most ill patients. However, the authors concluded that the current evidence is too weak to advocate widespread prophylactic use of vitamin C to prevent pneumonia in the general population, and further studies are needed to clarify the population that could have a benefit from vitamin C use. The effect of vitamin C on upper respiratory tract infections, such as the common cold, has also been studied in several trials. Vitamin C supplementation significantly decreases the incidence and the severity of the common cold in people under heavy physical stress [67,68]. In a randomised controlled pilot study, Garaiova et al. have shown a significant reduction in the incidence and duration of upper respiratory tract infection, but no significant differences were observed in the incidence rate ratio or duration of lower respiratory tract infection [69]. A recent meta-analysis comparing vitamin C with placebo demonstrated that administration of extra doses of vitamin C at the onset of a common cold could help reduce the duration by about half a day, shorten the time confined indoors, and relieve the symptoms of a common cold [70].

With the COVID-19 outbreak, vitamin C could play a role in preventing and treating the severe respiratory viral infection caused by SARS-CoV-2. The potential beneficial effect of vitamin C supplementation could be expected also from the depleted vitamin C levels that are present during a severe infection and in critically ill patients [71]. A recent randomised clinical trial (CITRIS-ALI) demonstrated that 96 hours’ infusion of vitamin C compared with placebo in patients with sepsis and ARDS did not improve the primary outcome of organ dysfunction scores, but significantly reduced mortality and significantly increased ICU-free days to 28 and hospital-free days to 60 [72].

Recently, Diao et al. retrospectively reviewed the numbers of total T cells, CD4^+^, CD8^+^ T cell subsets in a total of 499 COVID-19 patients and found a significantly reduction of T cells counts. Furthermore, they demonstrated a state of T cell dysfunction (T cell exhaustion) following SARS-CoV-2 infection [73]. Thus, the possibility that vitamin C affects viral respiratory tract infections, also supporting the viral clearance mediated by T cells, could encourage further studies aimed to investigate the role of vitamin C for prevention and treatment of COVID-19 disease.

## 5. Vitamin D

### 5.1. Metabolism and Functions

Vitamin D is a fat-soluble hormone that is mainly synthesized in the skin after exposure to ultraviolet rays from sunlight (in the form of vitamin D3), and, to a lesser extent, is derived from dietary intake in the form of either vitamin D2 or D3 (the main sources of vitamin D are fatty fish, fish oils, egg yolks, cheese, and vitamin D-fortified foods). After vitamin D is produced in the skin or absorbed through the gastrointestinal tract, it is transported to the liver by vitamin D-binding protein (VDBP). In the liver vitamin D is converted to 25 hydroxy vitamin D (25(OH)D), which is monitored to evaluate vitamin D status because of its half-life of 2–3 weeks. Next, 25 hydroxy vitamin D is transported to the kidneys, where it is finally converted to its active form, 1,25 dihydroxyvitamin D (1,25(OH)2D).

The actions of 1,25(OH)2D are mediated through ligation with a nuclear vitamin D receptor (VDR), leading to the regulation of the transcription of over 1000 target genes. VDR is widely distributed in many different cells and tissues, including the immune system. VDR gene polymorphisms, located on chromosome 12q13.1, have been associated with higher prevalence of respiratory infections [74,75,76,77]. One of the main roles of vitamin D is to maintain calcium homeostasis by promoting calcium absorption in the intestine and reabsorption in the kidneys and stimulating bone remodeling by increasing osteoclasts number. This effect was the first to be discovered, studying the causes of rickets and osteomalacia, but now it is thought that vitamin D has physiological effects much broader that its role in mineral homeostasis and bone function [78], including regulation of immunity, fetal development [79], and pulmonary function [80]. In addition, vitamin D can also induce cathelicidin in gastrointestinal epithelium [81] and plays a role in controlling gastrointestinal infections [82].

For the purpose of this review, we focused on the effects of vitamin D in modulating the immune system. Several mechanisms have been described [83]: firstly, vitamin D was found to induce the production of antimicrobial peptides such as cathelicidin and human beta-defensin from immune system cells such as neutrophils and macrophages and from epithelial respiratory cells [81,82,83,84,85,86,87]. Vitamin D also enhances the antimicrobial activity of macrophages by increasing TLR and CD14 expression [88], autophagy [89,90], and the activity of NADPH-dependent oxidase [91]; it also promotes the migration of dendritic cells to lymphoid organs where they can present antigens to T cells [92]. On the other side of the coin, vitamin D can also inhibit the production of pro inflammatory cytokines, which might appear counterproductive [93]; it is known, however, that the pathogenicity of respiratory viruses, including SARS-CoV2, can be linked to hypercytokinemia up to the so-called “cytokine storm” [94,95,96,97,98]. This immunoregulatory effect of vitamin D can thus be beneficial to the host while facing a viral infection.

It has been reported during influenza A infection that IFN-beta, tumor necrosis factor (TNF)-alfa, IL-8 and IL-6 in the lungs were reduced in response to treatment with vitamin D [99]; during RSV infection the NfkB inhibitor was induced [100]; similar immunomodulatory effects were also described during Dengue infection [101]. Vitamin D can suppress excessive activity of IFN gamma-activated macrophages [102]; decrease macrophagic cytokines release through upregulation of MKP-1 [103]; reduce the production of metalloproteinase MMP-9 in keratinocytes, whose excessive and potentially harmful activity is induced by TNF alfa during hyperinflammation [104].

Vitamin D can also regulate FOXP3 expression in T cells, thus inducing the differentiation of this cells to FOXP3+ T regulatory cells (T reg), which have an immunosuppressor activity [105,106], and can promote the secretion of anti-inflammatory IL-10 from T cells [107]. It was also reported in a placebo-controlled trial on healthy adults that high dose vitamin D supplementation significantly increased the frequency of circulating Tregs [108].

### 5.2. Vitamin D Status

There is currently no definitive consensus regarding the optimal concentration of vitamin D in children and adults. Most authors define vitamin D in normal range from 30 to 100 ng/mL, which might be the optimal range to ensure its immunoregulatory effects; insufficient between 20 and 29 ng/mL, and deficient if serum levels are <20 ng/mL (50 nmol/L), since this level is necessary to maintain optimal bone mineralization and calcium homeostasis in 97.5% of the population [109,110]. Severe vitamin D deficiency is defined as <10 ng/mL; below this cut-off, the risk of developing rickets is very high. Concentrations >100 ng/mL may instead be harmful, although toxicity is more commonly seen over 200 ng/mL.

A 2016 study [111] combined data from 14 European population studies, including children, adolescents, and adults, and found an estimated prevalence of insufficient vitamin D levels of 13% in the general population. The prevalence according to age in pediatric populations varied from 4%–7% (1–6 years), 1%–8% (7–14 years) and 12%–40% (15–18 years). Italian data usually regards smaller populations and reports a high prevalence of vitamin D deficiency and insufficiency. A 2014 study from Stagi and colleagues found a 30% prevalence of vitamin D insufficiency in Italian children and adolescents and a 58.7% prevalence of vitamin D deficiency [112]. In the same year, Vierucci and colleagues reported a prevalence of 32.3% insufficiency and 49.9% deficiency [113]. Cadario et al. [114] described in Italian newborns a high frequency of vitamin D deficiency (40.1%) and severe deficiency (38%).

### 5.3. Recommended Daily Allowance and Supplementation

In Italy, the currently recommended daily allowance for vitamin D is 10 mcg (400 IU) for infants up to 12 months of age, and 15 µg (600 IU) for children and adolescents [115]. Vitamin D prophylaxis is recommended to all newborns and infants up to 12 months of age, regardless of their being formula or breast-fed [110,116], as also recommended by various others international Scientific Societies [117,118,119,120]. A higher dose is recommended for preterm infants over 1500 gr of weight (600–800 IU/day); for very low birth weight (VLBW) newborns below 1500 gr an intake of 200–400 UI is recommended.

Vitamin D supplementation is also recommended for children and adolescents with risk factors (obesity, reduced sunlight exposure, intestinal malabsorption, chronic hepatic or kidney disease, chronic therapies such as anticonvulsants, ketoconazole, etc.), at the dose of 600 IU/die up to 1000 IU/die in the presence of multiple risk factors [110]. Other societies recommend systematical supplementation of vitamin D during winter months [121,122,123]. In Italy, despite a high prevalence of hypovitaminosis D, there is currently no indication to conduct routine testing in healthy children and adolescents without known risk factors, nor to routinely supplement vitamin D. In case of detection of vitamin D insufficiency (<20 ng/mL), it is recommended to administer a higher dose of vitamin D (2000 IU/day for 6–8 weeks).

### 5.4. Vitamin D against Lower Respiratory Tract Infections

Considering the above-mentioned role of vitamin D in modulating the immune response, many studies focused on the link between vitamin D and viral infections. In this review, we focused on the present knowledge on the relationship between vitamin D and lower respiratory tract infections (LRTI) in children, since they are a leading cause of morbidity and mortality worldwide, especially in developing countries and in children younger than 5 years. We searched PubMed using keywords such as “vitamin D” and “lower respiratory tract infections” or “viral infections,” focusing on studies on pediatric populations, including both observational studies and clinical trials. Numerous studies investigated the association between low levels of 25-hydroxyvitamin D and increased susceptibility to LRTI in childhood, as listed in Table 4 [124,125,126,127,128,129,130,131,132,133,134,135,136,137,138,139,140,141,142,143,144,145,146].

Since 1997, it has been shown in developing countries, where malnutrition and micronutrient deficiency were more common, that the incidence of pneumonia was higher in children with rickets [124,126,131], and treatment failure was seen more frequently in rachitic children [132]. Similar studies were conducted worldwide, evaluating the circulating levels of vitamin D in children with LRTI and in controls: several studies found that lower vitamin D levels were associated with higher risk of developing an acute respiratory tract infection [125,130,132,133,135,139,144], or were linked to a more severe course of illness [136,142], with more frequent need for oxygen supplementation, ventilation support [134], or increased risk of intensive care unit (ICU) admission and longer hospital stay [143].

Some studies showed contrasting evidence and found no difference in vitamin D status in LRTI patients vs. controls [129,137,141]. A 2016 study conducted in Hong Kong on children and adults found no significant association between lower levels of vitamin D and higher incidence of influenza virus infections [140]. McNally and colleagues [127] found no significant difference in the frequency of vitamin D deficiency between the LRTI group and healthy controls but evidenced that vitamin D levels were significantly lower in patients admitted to PICU. A 2015 American study [138] found no difference in the duration of hospitalization and in the severity of the disease between deficient and nondeficient children. As highlighted by the authors themselves, some of these findings might be explained by the different vitamin D status in different countries around the world, some implementing extensive vitamin D supplementation programs, and some still struggling with malnutrition and micronutrients deficiencies. Roth and colleagues [130] underlined how average vitamin D concentration varies through studies from different regions of the world, ranging from 22.8 nmol/L–equal to 9.12 ng/mL-in India and Turkey [125,145], 29.2 nmol/L in Bangladesh [128], to 77.2–81 nmol/L–equal to 30.8–32.4 ng/mL-in Canada [127,129].

Several studies also focused on the link between maternal vitamin D status during pregnancy and/or cord blood vitamin D levels and respiratory tract infections in infants. Higher vitamin D levels were found consistently associated with reduced risk of LRTI in infants worldwide [145,146,147,148,149,150,151,152,153,154], as described in Table 5.

These observational findings have laid the foundation for clinical trials of vitamin D supplementation for treatment or prevention of childhood respiratory tract infections, as shown in Table 6a,b [155,156,157,158,159,160,161,162,163,164,165,166,167,168,169,170].

These studies differ in many aspects, such as geographical location, baseline vitamin D level before the intervention, and dose and timing of vitamin D supplementation, and this heterogeneity leads to sometimes contradictory results.

A few studies investigated the effect of a short-term, high-dose vitamin D supplementation, which is the most practical administration scheme, but mostly found that these regimens were not significantly beneficial. Manaseki-Holland and colleagues evaluated the administration of a single high-dose (100,000 IU) vitamin D supplementation in addition to routine pneumonia treatment in children in Kabul, and evidenced a lower risk of recurrence in the intervention group but no difference in the time needed to recovery from the first infection [155]. Furthermre, a smaller study from Pakistan described a lower recurrence of pneumonia in children supplemented with a single dose of vitamin D [160]. Similar trials from Choudhary et al. [157] and Gupta et al. [165] tried a short-term vitamin D supplementation in Indian children with severe pneumonia: the first study supplemented vitamin D (1,000–2,000 UI/day) for 5 days, and the second 100,000 UI in a single dose; in both studies the authors did not evidence significant beneficial effects in the resolution of pneumonia in the intervention group. Gupta et al. evidenced only a slightly quicker resolution of the severe respiratory distress (1 h) in the intervention group, which might not be clinically relevant. Similar results were reached in 2017 by Somnath and colleagues [166], who investigated the efficacy of a single high dose of vitamin D in the treatment of children hospitalized with ALRI, and found it did not influence the duration of hospital stay nor the secondary outcomes (mortality, PICU admissions, complications, etc.). A supplementation of 50,000 IU/day for 2 days was tried in Iran in children with pneumonia and it did not influence the severity of symptoms, however the study reported a lower duration of antibiotic use in the intervention group [166]. Contrasting evidence was found in a 2015 Egyptian trial on children hospitalized for bronchiolitis [161], where the administration of vitamin D 100 IU/kg/day for 5 days was associated with a significant improvement in the duration of hospitalization and time taken to improve oral feeding. The efficacy of a high dose, short-term supplementation of vitamin D in preventing respiratory tract infections was also analyzed in 2012 by Manaseki-Holland and colleagues [157], who found 100,000 IU supplementation every 3 months ineffective in reducing the incidence of pneumonia, and later in 2019 by Singh et al. [169], who achieved similar results with a 300,000 IU supplementation every 3 month. Overall, the administration of a bolus dose or short-term supplementation of vitamin D did not demonstrate a consistent efficacy in treating nor in preventing LRTI [171], although there is, at times, conflicting evidence on the matter.

More promising results were reached using daily or weekly administration of vitamin D for longer periods of time. A 2010 Japanese study found that daily administration of vitamin D (1,200 IU/die) to schoolchildren during winter months reduced the incidence of influenza A infections [156]. In 2012, Camargo and colleagues [159] investigated the administration of vitamin D-fortified milk during winter months in Mongolian children, and reported significantly lower RTI episodes during the study period. A Chinese 2019 prospective study analyzed a cohort of infants recording whether they received vitamin D daily supplementation (400–600 UI/die) up to 6 months of age, and reported the median time of the first RTI episode, which ended up being 60 days in infants without supplementation and longer than 6 months in infants with supplementation [168].

The right dosage to achieve a protective effect on respiratory infections is yet to be established. Different studies evaluated different doses, ranging from 400 to 2000 UI/die. We found two studies that compared a lower vs. a higher dose of daily vitamin D supplementation, both reporting better results in the higher dose group.

In 2015 Grant and colleagues analyzed the supplementation of vitamin D to pregnant women and to their infants up to 6 months of age, comparing two regimens: 1000 IU to the mothers and 400 IU to the infants vs. 200–800 IU, and found a lower proportion of children made a primary care visit for respiratory infections up to 18 months of age in the higher dose group [163]. A 2018 Chinese study tested the efficacy of vitamin D in preventing influenza A, comparing a low dose scheme (400 IU/day) vs. a high dose one (1200 IU/day) for 4 months, and reported less frequent infections in the high dose group [167].

For the purpose of this review, we focused on studies on LRTIs, even though similar studies were also conducted on the prevention of upper airways infections; a large study conducted on the TARGet kids! research network in Toronto (Canada) led to different results, reporting that a high dose (2000 IU/day) was not more effective than a standard dose (400 IU/day) in preventing upper respiratory tract infections in children [172]. We also found two studies reporting negative results with vitamin D daily/weekly supplementation: the first, conducted in 2014, tested a daily supplementation of vitamin D 2000 IU/day for 2 months to Japanese high school students, and found no efficacy in lowering the overall incidence of influenza A [160]; the second, conducted in 2019 in Vietnam, analyzed a 14,000 IU/week supplementation of vitamin D to children and adolescents for 8 months, which was unable to prevent influenza infection during the flu season, but moderately reduced the incidence of other respiratory viral infections [172]. In this population, the authors reported a mean baseline vitamin D of 65 nmol/L (26 ng/mL), which might be one of the reasons why a further vitamin D supplementation did not lead to the expected results. In the previously cited studies, the baseline vitamin D status is not always reported; where it is known, it is usually lower, from 7 ng/mL, equal to 17.5 nmol/L [153], to 43 nmol/L, equal to 17.2 nmol/L [167].

In conclusion, there is evidence on the role of vitamin D in regulating the immune response to viral infections, and data from most observational studies confirm an association between lower vitamin D levels and increased susceptibility to respiratory infections. Clinical trials overall show that daily or weekly supplementation of vitamin D is more beneficial in preventing LRTI than bolus or short-term administration, as confirmed by a 2017 meta-analysis by Martineau and colleagues [171], though more research will be needed to fully determine when and how vitamin D should be supplemented. Vitamin D supplementation did not appear to be effective in treating existing infections in pediatric trials, as also described in a 2018 review from Das and colleagues [173]. The different results reached in the above-mentioned studies might be due to the heterogeneity in the baseline vitamin D status of the observed populations; it is also possible that vitamin D receptor’s polymorphisms affect the daily vitamin D requirements of different individuals. Future studies might better clarify which patients will benefit from vitamin D supplementation and which ones will not, which is the best dose to administer in each case, and whether vitamin D status should always be tested before intervention.

### 5.5. New Perspectives: Is There a Potential for Vitamin D Supplementation in Preventing COVID-19?

At the time of writing (3 July 2020), the COVID-19 pandemic has claimed over 500,000 lives worldwide with over 11 million confirmed infections. Different regions of the world have been differently affected by the pandemic, with Northern Italy setting an unfortunate record for incidence and mortality. Different factors might explain these geographical variations, such as the earlier spread of the virus in certain countries or the different preventive measures adopted, the different climates and air-pollution levels, or the different age-composition and social proximity of the communities. A North–South gradient in COVID-19 distribution has been noticed [174,175,176]. Areas along a latitude of 30–50° N with similar low-humidity, temperate weather, showed significant community spread of COVID-19 [177]. Marik and colleagues calculated the case-fatality rate in each state of the US and found increasing mortality with increasing latitude (>40° N) [178]. More recently, another study reported a highly significant, positive correlation between lower death rates and a country’s proximity to the equator [179]. Rhodes et al. described that more northerly countries are currently showing relatively high COVID-19 mortality, with an estimated 4.4% increase in mortality for each 1-degree latitude north of 28 degrees North [180].

Vitamin D deficiency is less common in countries where the sun exposure is consistent throughout the year or where the use of vitamin D fortified food is widespread. Various authors suggested that vitamin D deficiency might play a role in the variability of COVID-19 impact on different countries [175,176,177,181]. Ilie and colleagues searched literature for mean vitamin D level in each country and observed a negative correlation between vitamin D levels and number of COVID-19 cases and deaths [181]. Ali described a significant negative correlation between mean vitamin D levels and COVID-19 cases per one million population in European countries, as of 20 May 2020 [182].

Moreover, a wide variation in the severity of SARS-CoV2 infection’s clinical presentation has been noticed, ranging from absent or minimal symptoms to critical conditions and death. To date, although some risk factors have been identified (age, co-morbidities, etc.), it is not yet completely understood why some patients develop more severe symptoms than others. Considering our knowledge on the role of vitamin D in modulating the immune system and in inhibiting a hyper activation of the inflammatory response, together with data from observational and clinical studies on vitamin D supplementation, various authors have also suggested a potential role of vitamin D in reducing the severity of the disease [183,184,185,186,187]. Vitamin D is especially known for its ability to reduce the “cytokine storm” that contributes to the pathogenesis of various viral infections, including COVID-19 [188].

To date, we only have preliminary observations regarding the association of vitamin D deficiency and frequency and severity of COVID-19; the above mentioned study from Ilie and colleagues found a correlation between mean vitamin D levels in each country and COVID-19 cases and deaths [181]; D’Avolio and colleagues investigated vitamin D concentrations in a small cohort of 107 patients with a positive naso-pharyngeal swab for SARS-CoV2 in Switzerland, and found significantly lower vitamin D levels in patients than in controls with negative swabs [187]; Lau et al. described a high frequency of vitamin D insufficiency (84.6%) in COVID-19 patients admitted to ICU in New Orleans, with a 100% frequency in patients younger than 75 years [188].

Interestingly, a recent pilot study demonstrated that administration of a high dose of 25-hydroxyvitamin D significantly reduced the need for intensive care unit treatment of patients requiring hospitalization due to proven COVID-19 [189]. Calcifediol seems to be able to reduce severity of the disease, but larger trials with groups properly matched will be required to show a definitive answer.

## 6. Omega-3 Fatty Acids

### 6.1. Metabolism and Functions

Omega-3 fatty acids are a family of polyunsaturated fatty acids (PUFAs) characterized by the presence of a double bond at the omega−3 carbon atom. The simplest omega-3 fatty acid is α-linolenic acid (18:3n-3), which is synthesized from the omega-6 fatty acid linoleic acid (18:2n-6) by desaturation, catalysed by delta-15 desaturase.

Linoleic acid (18:2n-6) and α-linolenic acid (18:3n-3) are essential fatty acids (EFAs), meaning that they must be obtained from the diet. Indeed, they are synthesized by plants and cannot be synthesized sufficiently by the human organism [190]. However, animals can metabolize α-linolenic acid by further desaturation and elongation to yield eicosapentaenoic acid (20:5n-3; known as EPA) and docosahexaenoic acid (22:6n-3; known as DHA). It is important to note that the same enzymes are employed by omega-6 fatty acids for their metabolic pathways, which leads to the production of arachidonic acid. This means that α-linolenic acid is a competitive inhibitor of linoleic acid metabolism and vice versa [191]. However, it has been demonstrated that the conversion to EPA and DHA is generally poor in humans, with reported rates of less than 15%. Therefore, these fatty acids must be supplied with food [192]. Alfa-linolenic acid is present in plant oils, DHA and EPA are present in fish, fish oils, and krill oils.

Omega-3 fatty acids play important roles in the body as components of the phospholipids that form the structures of cell membranes. Furthermore, they provide energy for the body and are used to form eicosanoids, exercising several functions in the body’s cardiovascular, pulmonary, immune, and endocrine systems [189]. Both omega-3 and omega-6-derived metabolites have important immune-regulatory functions [193]. PUFAs represent substrates for the enzymatically production of molecules that play an important role in the resolution of inflammation, named specialized pro-resolving mediators (SPMs) [194,195]. These molecules are distinct from immunosuppressive agents because they contribute to the inflammatory response resolution but also display antimicrobial action promoting host defence [196]. SPMs derived from omega-3 fatty acid (EPA and DHA) are classified as resolvins, protectins, and maresins. These pro-resolving mediators are important in supporting immune cell functions to neutralize and eliminate pathogens and play a crucial role in promoting the resolution of inflammation [197].

Omega-3 fatty acids metabolites resolvins are effective in inhibiting neutrophil migration, reducing further neutrophil entry in the inflammation site [198,199]. Furthermore, SPMs exercise a potent anti-inflammatory action, also reducing tissue neutrophil activation and preventing tissue damage [197,200]. In order to obtain tissue resolution of inflammation, it is essential the clearance of apoptotic neutrophils and protectins stimulate phagocytosis of apoptotic cells mediated by macrophages [201,202,203,204,205]. Furthermore, SPMs stimulate natural killer cells to trigger granulocyte apoptosis, accelerating the clearance of apoptotic polymorphonuclear leukocytes [206]. The anti-inflammatory response is promoted by SPMs also by dampening cytokine production. A study of Ariel et al. suggests that pro-resolving mediators upregulate CCR5 expression on apoptotic, activated T cells, thus sequestering pro-inflammatory cytokines, and promoting the resolution of the inflammation [207].

### 6.2. Omega-3 Fatty Acids Status

Plasma and serum fatty acid values can vary significantly based on an individual’s most recent meal, so they do not reflect long-term dietary intake. However, omega-3 status can be valued by calculating the percentage of the total serum phospholipid fatty acids. Although a normal range is not established, mean values for serum phospholipid EPA plus DHA are about 3–4% [206]. Omega-3 status could also be assessed analysing erythrocyte fatty acids. Harris and von Schacky proposed the “omega-3 index”, which represents the content of EPA plus DHA in red blood cells membranes, expressed as a percentage of total erythrocyte fatty acids, and reflects better long-term intake of EPA and DHA [207,208]. EPA and DHA are about 3–5% of erythrocyte fatty acids in Western populations with low fish intakes [209]. Moreover, the recent discovery of novel dietary omega-3 and omega-6 lipid-derived metabolites-such as resolvin, protectin, maresin, 17,18-epoxy-eicosatetraenoic acid, and microbe-dependent 10-hydroxy-cis-12-octadecenoic acid, and their potent biologic effects on the regulation of inflammation, have initiated a new era of nutritional immunology [210]. It has been shown that a synergy between omega-3 fatty acids and gut microbiota enhances the efficacy of immune checkpoint inhibitors [211].

### 6.3. Recommended Daily Allowance and Supplementation

Since insufficient data are available to establish an estimated average requirement (EAR), the EFSA panel on Dietetic Products, Nutrition, and Allergies (NDA) indicated adequate intake (AI) for adults of 250 mg for eicosapentaenoic acid plus docosahexaenoic acid based on considerations of cardiovascular health. For older infants (>6 months of age) and young children, below the age of 24 months, was proposed an adequate intake of 100 mg docosahexaenoic acid. For the age period 2 to 18 years, the AI proposed for the adult population should be considered suitable [212].

### 6.4. Omega-3 Fatty Acids Supplementation against Viral Infection

As mentioned above, the omega-3 fatty acids play a crucial role in the resolution of inflammation induced by infections, including in the respiratory tract [196]. Table 7 summarizes the main studies in which were investigated the link between the omega-3 fatty acids supplementation and respiratory infections/illness, and the potential role in improving the acute lung injury and acute respiratory distress syndrome (ARDS) [213,214,215,216,217,218,219,220,221,222,223].

Some studies investigated the effects of the omega-3 fatty acids supplementation on infant morbidity, particularly caused by respiratory tract infections, wheezing, and asthma. Imhoff et al. showed that DHA supplementation during pregnancy decreased the occurrence of colds in children at 1 month and influenced illness symptom duration [213]. Pastor et al. in a multicenter, prospective, open-label observational study, which included 1342 infants, showed a higher incidence of bronchiolitis in control versus groups who received omega-3-supplemented formula [214]. In contrast, in another study aimed to valuate the effect of neonatal DHA supplementation, the hospitalisation for lower respiratory tract problems in the first 18 months for preterm infants was not reduced [215]. A randomized controlled, trial which included 736 pregnant women and a total of 695 children, showed that the risk of persistent wheeze or asthma was reduced by approximately 7% in the first 5 years of life among children of women who received daily supplementation with omega−3 PUFA (EPA/DHA) during the third trimester of pregnancy. It is notable that this effect was most prominent among children of women with low EPA and DHA blood levels at randomization. Furthermore, supplementation was also associated with a reduced risk of infections of the lower respiratory tract [216].

Some studies have demonstrated the effect of omega-3 supplementation also on children’s morbidity, particularly reducing the episodes of upper respiratory tract infections [217,218]. Malan et al. in a randomized, double-blind, placebo-controlled trial, which included 321 South African children with iron-deficiency, showed that iron supplementation was associated with an increased morbidity, mostly respiratory, but when given in combination with DHA/EPA, this increase in morbidity was prevented. Authors suggested that this effect could be explained by the DHA- and EPA-mediated protection against iron-induced oxidative stress and the improved resolution of inflammation [219].

It has been shown that severe COVID-19 could manifest as a hyperinflammatory syndrome (secondary haemophagocitic lymphohistiocytosis), which is characterized by an important hypercytokinaemia (cytokine storm) with multiorgan failure and ARDS in approximately 50% of patients [220]. Several studies have been conducted to determinate if omega-3 fatty acids DHA and EPA could modulate systemic inflammatory response and affect plasma cytokine production. Thienprasert et al., in a randomized controlled trial, demonstrated that consumption of omega-3 PUFAs was associated with fewer episodes and shorter duration of illness (mainly upper respiratory tract) and with a significantly lower concentration of TGF-beta1 concentration compared with the placebo group [221]. Two randomized controlled trials, aimed to determinate if omega-3 fatty acids could modulate the systemic inflammatory response, improving the outcomes in patients with acute lung injury, have shown that in the intervention groups there was not a reduction of the biomarkers of systemic inflammation and pulmonary outcomes did not improve [222,223]. In a recent systematic review, Dushianthan et al. have reported a significant improvement in blood oxygenation and in the duration of ventilator days and ICU length of stay in patients with ARDS who received nutrition containing antioxidants and rich in EPA and DHA, although there was a low quality of evidence [224].

These findings supported also by results of animal studies [225,226,227], may suggest a potential role for EPA and DHA in reducing the lung injury supporting the resolution of inflammation, probably via the production of SPMs [207]. However, further trials are needed to support this hypothesis.

## 7. Zinc

### 7.1. Metabolism and Functions, Recommended Daily Allowances

Zinc is an essential trace element for humans, required for the function of numerous enzymes and transcription factors. It plays a key role in regulating the function of both the adaptive and the innate immune system [14,228,229]. Dietary sources of zinc are animal products such as meat, fish, eggs, and dairy, but it is also contained in whole grains, nuts, and legumes. Zinc from animal sources has higher bioavailability compared to zinc derived from plant products. Non-digestible plant ligands such as phytate, some dietary fibers, and lignin chelate zinc and inhibit its absorption.

Zinc is absorbed throughout the digestive tract through specific transporters, such as ZIP4 (SLC39A4), whose mutation is responsible for the rare, lethal autosomal-recessive inherited *acrodermatitis enteropathica*.

Zinc deficiency is estimated to affect billions of people worldwide, especially the elderly and children in developing countries, pregnant women, vegan, and vegetarians. Zinc is considered deficient if plasmatic levels are below 60 mcg/dL. In Italy, the recommended daily allowance for zinc is 3 mg/day for infants below 12 months of age, then it raises gradually to a recommended intake of 9–12 mg/day for adolescents and adults [230].

Zinc’s effect on the immune system is complex; it can both enhance and inhibit different immune functions to reach a correct balance between pro and anti-inflammatory effects through various mechanisms. A correct intake of zinc is essential to limit the overproduction of inflammatory cytokines: in vitro and human studies show that zinc deficiency is associated with an increased inflammatory response and excessive release of pro inflammatory cytokines such as IL-2, IL-6, and TNF-alfa, regulated through the NF-κB signaling pathway [230,231,232,233,234,235,236,237]. Zinc also enhances the number of inducible regulatory T cells [238,239,240,241]. Another important role played by zinc is the maintenance of membrane barrier integrity, which is essential in the pulmonary and intestinal epithelia that constitute the first barrier to protect the organism from pathogens. [242,243,244]. Zinc supplementation is also effective in decreasing oxidative stress [245,246], in shortening the duration of cold symptoms in adults [247], and was found to have a direct antiviral effect on RSV [248], Dengue virus [249], and coronaviruses [250]. Lastly, some authors suggested that a combination of chloroquine with zinc might enhance chloroquine’s toxicity on viruses [249,250,251,252]. Te Velthius et al. [249] reported that the combination of Zn2+ and zinc-ionophores like pyrithione can increase the intracellular Zn2+ concentration, and thus inhibits the replication of SARS-coronavirus (SARS-CoV) and equine arteritis virus in cell culture.

### 7.2. Zinc Supplementation in Treating Viral Infections

Considering the known effects of zinc in regulating the immune system, trials have been conducted to investigate the efficacy of zinc in treating respiratory illnesses (Table 8) [253,254,255,256,257,258,259,260,261,262,263,264,265,266,267].

In a 2018 study, serum zinc levels were found to be significantly lower among pneumonia pediatric patients admitted to PICU compared with patients admitted to other wards; there was a statistically significant decrease in zinc level in critically ill children complicated by sepsis, mechanically ventilated and fatal cases [252]. Some studies reported similar duration of hospital stay, time to symptoms resolution, and risk of treatment failure in the intervention and in the control group [254,255,256,258,259,262,264,265], while others described partially positive results. Basnet and colleagues [260] found that zinc recepients recovered slightly faster than controls, although the difference was not statistically significant; Sempertegui et al. [263] evidenced that a higher basal zinc concentration was associated with faster resolution of chest indrawing, although there was no difference in time needed to fully recover nor in the risk of treatment failure; Mahalanbis et al. found a reduced duration of ALRI symptoms in boys, but not in girls [253].

Other trials evidenced instead positive results with zinc supplementation in acute respiratory infections: in 2011, Valavi et al. [257] described a faster resolution of symptoms in zinc-supplemented children; in 2012, a lower mortality rate was evidenced in Uganda in children who received zinc supplementation during the acute infection [261]; in 2019, Acevedo-Murillo and colleagues [266] reported a quicker improvement in the clinical status of pneumonia pediatric patients receiving zinc supplementation; in a Thailandese 2019 trial, zinc supplementation was associated with a shorter hospital stay and quicker resolution of symptoms [267].

Overall, the available data does not conclusively assert the efficacy of zinc in treating an existing acute lower respiratory illness in children, as also described in a 2012 meta-analysis from Das and colleagues [268]; more studies are needed to definitively establish if, and at what dose, zinc should be supplemented to children during an acute respiratory infection.

### 7.3. Zinc Supplementation in Preventing Viral Infections

From the early 2000s, various studies were conducted, mainly in children from lower socio-economic settings, to establish whether daily or weekly zinc supplementation could help in preventing respiratory tract infections (Table 9) [269,270,271,272,273,274,275,276,277,278,279,280,281].

Different studies reported a reduced incidence of respiratory infections in the zinc receiving group [269,271,272,273,280]. In 2007, Sazawal et al. reported a slight reduction in the relative risk of all-cause mortality in children supplemented with zinc in Zanzibar [277]. Other studies found instead that zinc supplementation had no significant effect on the frequency of respiratory infections [274,275,276,278,279,281]. Some of these trials used a lower daily dose of zinc, which might be one of the reasons why the supplementation was less effective; however, both negative and positive results were reported with different zinc dosages, from 5 up to 30 mg/day.

Overall, different reviews and meta-analysis confirmed the efficacy of zinc supplementation in preventing respiratory illnesses: Aggarwal et al. reported that zinc supplementation for more than 3 months significantly reduced the frequency and severity of diarrhea and respiratory illnesses [282]; in 2010, Roth et al. found that routine zinc supplementation reduced the incidence of childhood ALRI [283]; in 2011, Yakoob and colleagues described a reduction in diarrhea and pneumonia mortality in children from developing countries who received zinc supplementation for over 3 months [284]; a 2016 systematic review also reported a statistically significant lower incidence of pneumonia (−13%) in children receiving zinc supplementation [285].

## 8. Conclusions

A well-functioning immune system is important to help reduce the risk of infections, including LRTIs. Currently published data confirmed the well-established role of micronutrients in supporting the immune system. The in vitro and observational studies, and clinical trials, reported in this review show the important role of vitamins A, C, and D, omega 3 fatty acids, and zinc in modulating the immune response. Vitamins and other micronutrients are understood to work together to support an effective immune system, based on a variety of mechanistic and clinical data. However, further studies are needed to evaluate nutrients’ synergistic effects in the immune response against viral infetions. Supplementation with vitamins, omega 3 fatty acids, and zinc appears to be a safe and low-cost way to support optimal function of the immune system, with the potential to reduce the risk and consequences of infection, including viral respiratory infections. Supplementation should be in addition to a healthy diet and fall within recommended upper safety limits set by scientific expert bodies. Therefore, implementing an optimal nutrition, with micronutrients and omega-3 fatty acids supplementation, might be a cost-effective, underestimated strategy to help reduce the burden of infectious diseases worldwide, including COVID-19. Nevertheless, it is important to recognize that nutritional supplementation will not necessarily prevent infections, or cure the disease, but may help decrease symptoms and facilitate recovery.

## Figures and Tables

**Table 1:** Dietary reference intakes recommended for vitamins A and C.

Micronutrient	Age Range	Recommended Dietary Allowance (RDA)	Upper Level (UL)
Vitamin A (μg retinol equivalents per day)	0–6 m	400	600
	7–12 m	500	600
	1–3 y	300	600
	4–8 y	400	900
	9–13 y	600	1700
	14–18 y	900 (male), 700 (female)	2800
Vitamin C (mg per day)	0–6 m	40	ND
	7–12 m	50	ND
	1–3 y	15	400
	4–8 y	25	650
	9–13 y	45	1200
	14–18 y	75–105 (male), 65–90 (female)	1800

From Sullivan KE and Buckley RH [1], revised. y, years; m, months; ND, not determined.

**Table 2:** Main studies on vitamin A supplementation against viral infections.

Study	Author	Study Population	Micronutrient (Dosage)	Results
Vitamin A and respiratory syncytial virus infection. Serum level and supplementation trial.	Kyran P 1996 [37]	Children RCT	Vitamin A (100,000 UI)	Lower mean vitamin A levels in RSV-infected children than in healthy control (*p* < 0.05). No significant difference in improvement in clinical outcomes.
Treatment of respiratory syncytial virus infection with vitamin A: a randomized placebo-controlled trial in Santiago.	Dowell SF 1996 [38]	Children RCT	Vitamin A (50,000 to 200,000 UI, dosed according to age)	More rapid resolution of tachypnea (*p* = 0.01). Shorter duration of hospitalization (*p* = 0.09).
Vitamin A therapy for children with respiratory syncytial virus infection: a multicenter trial in the United States.	Bresee JS 1996 [39]	Children RCT	Vitamin A (50,000 to 200,000 UI, dosed according to age)	Not significantly different in the number of days during which supplemental oxygen was required. Not significant difference in the number of days required to achieve normal respiratory rates.
Vitamin A supplements and diarrheal and respiratory tract infections among children in Dar es Salaam, Tanzania.	Fawzi WW 2000 [40]	Children RCT	Vitamin A (100,000 to 200,000 UI, dosed according to age)	Significantly higher risk of cough and rapid respiratory rate (*p* = 0.004) in treatment group.
Vitamin A for preventing acute lowe respiratory tract infections in children up to 7 years of age.	Chen H. 2008 [43]	Children from areas or with conditions correlated with a status of vitamin A deficiency. 10 RCTs	Vitamin A (6 studies were large-dose trials (100,000 UI o 200,000 UI) 4 studies were low-dose trials (5000 UI daily or 10,000 UI weekly or 45,000 UI every 2 months)	No significant effect on the incidence or prevalence of ALRI symptoms with vitamin A supplementation.
Vitamin A supplementation for prophylaxis or therapy in childhood pneumonia: a systematic review of randomized controlled trials.	Mathew JL 2010 [42]	Children 20 RCTs	Vitamin A (prophylaxis trial: >100,000 UI; therapeutic trials: 100,000 UI o 200,000 UI)	Neither prophylactic nor therapeutic benefit for childhood pneumonia.
Vitamin A supplementation every 6 months with retinol in 1 million pre-school children in north India: DEVTA, a cluster-randomized trial.	Awasthi S 2013 [32]	Children RCT	Vitamin A (200,000 UI 6-monthly)	Not significant mortality reduction.
Vitamin A supplementation for preventing morbidity and mortality in children from 6 months to 5 years of age.	Imdad A 2017 [33]	Children 42 RCTs	Vitamin A (large-dose trials: range of 50,000 UI to 200,000 UI, except for five studies: 3866 UI 3 times a week, 8333 UI once a week, 10,000 UI weekly and 250,000 UI 2 times a week)	12% reduction in all-cause mortality (RR 0.88 95% CI 0.83 to 0.93) in the interevention group. Not significant difference in ALRI-mortality. Not effect for vitamin A supplementation on ALRI incidence (only 2 trials reported ALRI prevalence, suggesting benefit for vitamin A supplementation).

ALRI, acute lower respiratory tract infection; CI, confidence interval; RCT, randomized controlled trial; RR, relative risk; RSV, respiratory syncytial virus.

**Table 3:** Main studies on vitamin C supplementation against viral infections.

Study	Author	Study Population	Micronutrient (Dosage)	Results
The effect of vitamin C on upper respiratory infections in adolescents swimmers: a randomized trial.	Constantini NV 2011 [68]	Children (12–17 years) RCT	Vitamin C (1 g/day for 3 months)	In the male swimmers duration (*p* = 0.003) and severity (*p* = 0.003) of URTI episodes were decreased.
Vitamin C for preventing and treating the common cold.	Hemilä H 2013 [67]	Adults and children 31 RCTs	Vitamin C (>0.2 g/day)	Significant reduction in the duration of common cold episodes. Decreased cold severity in Vitamin C group.
Vitamin C for preventing and treating pneumonia.	Hemilä H 2013 [65]	Adults 3 RCTs	Vitamin C	Significant reduction in pneumonia risk (>80%). Lower mortality in the vitamin C group vs. placebo group.
Vitamin C supplementation slightly improves physical activity levels and reduces cold incidence in men with marginal vitamin C status: a randomized controlled trial.	Schumacher SS 2014 [66]	Adults RCT	Vitamin C (1 g/day for 8 weeks)	Reduced cold episodes in young men with low to average vitamin C status (*p* = 0.04). Cold duration was reduced 59% in the vitamin C vs. placebo groups (*p* = 0.06).
Probiotics and vitamin C for the prevention of respiratory tract infections in children attending preschool: a randomised controlled pilot study.	Ďuračková Z 2015 [69]	Children RCT	Vitamin C (50 mg/day for 6 months)	Reduced incidence (*p* = 0.002) and duration (*p* = 0.006) of URTIs. No significant differences in the duration or incidence of LRTIs.
Extra dose of vitamin C based on a daily supplementation shortens the common cold: a meta-analysis of 9 randomized controlled trials.	Ran L 2018 [70]	Adults and children 9 RCTs	Vitamin C (4–8 g/day) administrated at the onset of cold	Reduced cold duration (MD = −0.56, *p* = 0.02). Relieved cold symptoms including chest pain (MD = −0.40, *p* = 0.03), fever (MD = −0.45, *p* = 0.009), and chills (MD = −0.36, *p* = 0.01).
Effect of vitamin C infusion on organ failure and biomarkers of inflammation and vascular injury in patients with sepsis and severe acute respiratory failure: the CITRIS-ALI randomized clinical trial.	Fowler AA 2019 [72]	Adults RCT	Vitamin C (IV 50 mg/kg every 6 h for 96 h)	Significant reduction in 28-day all-cause mortality (*p* = 0.03), and with significantly increased ICU-free days to day 28 (*p* = 0.03) and hospital-free days to day 60 (*p* = 0.04).

ICU, intensive care unit; LRTI, lower respiratory tract infection; MD, mean difference; RCT, randomized controlled trial; URTI, upper respiratory infection.

**Table 4:** Studies on the association between low levels of 25-hydroxyvitamin D and increased susceptibility to lower respiratory tract infections (LRTI) in childhood.

Study	Author	Country	Study Population	Results
Case-control study of the role of nutritional rickets in the risk of developing pneumonia in Ethiopian children.	Muhe et al., 1997 [124]	Ethiopia	500 children with pneumonia vs. 500 healthy controls	Higher incidence of rickets in children with pneumonia.
Association of subclinical vitamin D deficiency with severe acute lower respiratory infection in Indian children under 5 years.	Wayse et al., 2004 [125]	India	Children with severe ALRI vs. controls	Vitamin D levels >22.5 nmol/L associated with lower risk of severe ALRI.
The frequency of nutritional rickets among hospitalized infants and its relation to respiratory diseases.	Najada et al., 2004 [126]	Jordan	443 children hospitalized due to different causes	Higher risk of being admitted due to LRTI and significantly more prolonged hospital stay in children with rickets.
Vitamin D deficiency in young children with severe acute lower respiratory infection.	McNally et al., 2009 [127]	Canada	105 children <5 years with ALRI vs. healthy controls	Significantly lower vitamin D levels in children admitted to PICU.
Nutritional rickets and vitamin D deficiency–association with the outcomes of childhood very severe pneumonia: a prospective cohort study.	Banajeh et al., 2009 [128]	Yemen	152 children aged 2–59 months with pneumonia	Significantly more frequent treatment failure in rachitic children; vitamin D deficiency associated with day 5 hypoxemia <88%.
Vitamin D status is not associated with the risk of hospitalization for acute bronchiolitis in early childhood.	Roth et al., 2009 [129]	Canada	64 children aged 1–25 months with ALRI vs. healthy controls	Similar vitamin D concentrations among cases and controls.
Vitamin D status and acute lower respiratory infection in early childhood in Sylhet, Bangladesh.	Roth et al., 2010 [130]	Bangladesh	25 children aged 1–18 months with ALRI vs. 25 healthy controls	Significantly lower vitamin D in ALRI cases than in controls.
Frequency of nutritional rickets in children admitted with severe pneumonia.	Haider et al., 2010 [131]	Pakistan	137 children with severe pneumonia	High frequency of rickets (74% of cases).
Relationship between vitamin D levels and outcome of pneumonia in children.	Oduwole et al., 2010 [132]	Nigeria	24 children with pneumonia vs. healthy controls	Lower vitamin D levels in cases than in controls; increased complications frequency when lower vitamin D levels.
Low serum 25-hydroxyvitamin D levels are associated with increased risk of viral coinfections in wheezing children.	Jartti et al., 2010 [133]	Finland	children hospitalized for wheezing	Lower vitamin D level linked to higher risk of having a viral infection.
Serum vitamin D concentrations and associated severity of acute lower respiratory tract infections in Japanese hospitalized children.	Inamo et al., 2011 [134]	Japan	28 children with ALRI	Vitamin D deficiency (<15 ng/mL) correlates to the need for supplementary oxygen and ventilator management.
Vitamin D intake in young children with acute lower respiratory infection.	Leis et al., 2012 [135]	Canada	children with ALRI vs. controls	Children reporting a lower vitamin D intake were more likely to have ALRI.s
Correlation between serum vitamin D level and severity of community acquired pneumonia in young children.	Ren et al., 2013 [136]	China	103 children with CAP vs. healthy controls	Lower vitamin D levels in severe CAP cases than in mild CAP and controls.
The association between 25-dehydroxy vitamin D and lower respiratory infection in children aged less than 5 years in Imam Reza hospital, Bojnurd, Iran.	Khakshour et al., 2015 [137]	Iran	90 children hospitalized either for acute LRTI or for other reasons	Not significantly different vitamin D levels between the two groups.
Vitamin D Levels Are Unrelated to the Severity of Respiratory Syncytial Virus Bronchiolitis Among Hospitalized Infants.	Beigelman et al., 2015 [138]	USA	Children hospitalized with bronchiolitis	Similar duration of hospitalization and severity of the disease in deficient and non-deficient children.
Association of Vitamin D Deficiency with Acute Lower Respiratory Infection in Toddlers.	Narang et al., 2016 [139]	India	50 children hospitalized with ALRI vs. 50 healthy controls	Lower vitamin D levels in cases than in controls (mean level 20.4 ng/mL).
Serum 25-Hydroxyvitamin D Was Not Associated with Influenza Virus Infection in Children and Adults in Hong Kong, 2009–2010.	Xu et al., 2016 [140]	Hong Kong	Over 3000 children and adults	Vitamin D levels not significantly associated with frequency of influenza infections.
Evaluation of serum 25-hydroxy vitamin D levels in children with acute bronchiolitis.	Mahyar et al., 2017 [141]	Iran	57 children with bronchiolitis vs. 57 healthy controls	No significant difference between the 2 groups.
The effect of vitamin D deficency on the severity of bronchiolitis in infants.	Erol et al., 2017 [142]	Turkey	Children with bronchiolitis	Higher incidence of vitamin D deficiency in children with moderate or severe bronchiolitis.
Vitamin D Status at the Time of Hospitalization for Bronchiolitis and Its Association with Disease Severity.	Vo et al., 2018 [143]	USA	Over 1000 children hospitalized with bronchiolitis	Vitamin D deficiency correlates to increased risk of intensive care admission and longer hospital stay.
Association between serum 25-hydroxyvitamin D concentration and pulmonary infection in children.	Li et al., 2018 [144]	China	Children with pneumonia vs. healthy controls	Lower vitamin D levels in the pneumonia group (mean 19 ng/mL), especially in the pneumonia induced sepsis subgroup.

ALRI, acute lower respiratory tract infection; CAP, community-acquired pneumonia; LRTI, lower respiratory tract infection; PICU, pediatric intensive care unit.

**Table 5:** Levels of 25-hydroxyvitamin D during pregnancy and in neonates and risk of lower respiratory tract infections (LRTI) in infancy.

Study	Authors	Country	Study Population	Results
Association of subclinical vitamin D deficiency in newborns with acute lower respiratory infection and their mothers.	Karatekin et al., 2009 [145]	Turkey	25 newborns with LRTI admitted to NICU vs. healthy controls	Significantly lower vitamin D levels in affected newborns than in controls.
Cord blood vitamin D deficiency is associated with respiratory syncytial virus bronchiolitis.	Belderbos et al., 2011 [146]	Netherlands	156 healthy newborns	Lower cord blood vitamin D in neonates who developed RSV infections.
Maternal vitamin D status in pregnancy and risk of lower respiratory tract infections, wheezing, and asthma in offspring.	Morales et al., 2012 [147]	Spain	1724 pregnant women	Decreased LRTI in offspring of mothers with higher vitamin D.
Cord blood 25-hydroxyvitamin D levels and the risk of acute lower respiratory tract infection in early childhood.	Mohameet al., 2013 [148]	Egypt	206 healthy newborns	Increased risk of developing ALRIs in the first 2 years of life in newborns with lower cord blood vitamin D.
Prospective study of maternal mid-pregnancy 25-hydroxyvitamin D level and early childhood respiratory disorders.	Magnus et al., 2013 [149]	Norway	Pregnant women	Reduced risk of LRTI in offspring by age 36 months when maternal vitamin D was higher.
Cord blood 25(OH)D levels and the subsequent risk of lower respiratory tract infections in early childhood: the Ulm birth cohort.	Luczynska et al., 2014 [150]	Germany	777 healthy newborns	Increased risk of developing LRTIs in the first year of life in infants with vitamin D deficiency in cord blood.
Cord blood vitamin D and the risk of acute lower respiratory infection in Indigenous infants in the Northern Territory.	Binks et al., 2016 [151]	Australia	109 mother-infant pairs	Higher risk of hospitalization for ALRI in pairs with lower vitamin D levels in pregnancy, cord blood and infants’ blood.
Association of vitamin D deficiency with acute lower respiratory tract infections in newborns.	Dinlen et al., 2016 [152]	Turkey	30 newborns with ALRI and their mothers vs. healthy control pairs	Lower vitamin D levels in ALRI group than in healthy controls.
Low cord-serum 25-hydroxyvitamin D levels are associated with poor lung function performance and increased respiratory infection in infancy.	Lai et al., 2017 [153]	Taiwan	122 mother-infant pairs	Higher risk of RTI and poorer lung function in infants with lower vitamin D levels (maternal and cord blood).
Vitamin D Status in Neonatal Pulmonary Infections: Relationship to Inflammatory Indicators.	El-Kassas et al., 2019 [154]	Egypt	33 neonates with pneumonia vs. healthy controls	Lower levels of vitamin D in pneumonia patients.

ALRI, acute lower respiratory tract infection; LRTI, lower respiratory tract infection; NICU, neonatal intensive care unit; RSV, respiratory syncytial virus; RTI, respiratory tract infection.

**Table 6:** (**a**) Vitamin D supplementation for treatment childhood respiratory tract infections; (**b**) Vitamin D supplementation for prevention of childhood respiratory tract infections.

Study	Author	Country	Study Population	Dosage	Results
(**a**) Vitamin D supplementation for treatment childhood respiratory tract infections					
Effects of vitamin D supplementation to children diagnosed with pneumonia in Kabul: a randomised controlled trial.	Manaseki-Holland et al., 2010 [155]	Afghanistan	453 children with pneumonia	Single dose 100,000 IU	No significant difference in the number of days needed to recover. Lower risk of recurrence in the intervention group.
Vitamin D supplementation for severe pneumonia—a randomized controlled trial.	Choudhary et al., 2012 [157]	India	200 children with severe pneumonia	1000 IU if <1 y or 2000 IU if >1 y, once a day for 5 days	No beneficial effects on resolution of severe pneumonia.
Trial of vitamin D supplementation in infants with bronchiolitis: A Randomized, Double-Blind, Placebo-Controlled Study.	Saad et al., 2015 [161]	Egypt	89 infants with bronchiolitis	100 IU/kg/day for at least 5 days during hospital stay	Significant improvement in the duration of hospitalization and time taken to improve oral feeding.
Efficacy of vitamin D in children with pneumonia: a randomized control trial study.	Dhungel et al., 2015 [162]	Pakistan	200 children with pneumonia	Single dose 100,000 IU	Lower recurrence of pneumonia, similar duration of hospital stay.
The effects of vitamin D supplementation in respiratory index of severity in children (risc) of hospitalized patients with community-acquired pneumonia: a double-blind randomized clinical trial	Rahmati et al., 2016 [164]	Iran	Children hospitalized with pneumonia.	50,000 IU per day for 2 days	Lower duration of antibiotic use; other clinical characteristics were similar (fever, retractions, tachypnea, poor feeding, etc.).
Vitamin D supplementation for treatment and prevention of pneumonia in under-5 children: a randomized double-blind placebo-controlled trial.	Gupta et al., 2016 [165]	India	324 children with severe pneumonia	Single dose 100,000 IU	No significant difference in duration of hospitalization, complete resolution of symptoms and risk of recurrent pneumonia; slightly quicker resolution of severe respiratory distress (1 h).
Therapeutic effect of vitamin D in acute lower respiratory infection: A randomized controlled trial.	Somnath et al., 2017 [166]	India	154 children with ALRI	Single dose 100,000 IU	No significant difference in the duration of hospital stay nor in the secondary outcomes (mortality, PICU admissions, complications, recurrence, etc.).
Effect of Vitamin D Supplementation in the Prevention of Recurrent Pneumonia in Under-5 Children.	Singh et al., 2019 [169]	India	100 children with pneumonia	300,000 IU quarterly	No significant difference in ARI recurrence.
(**b**) Vitamin D supplementation for prevention of childhood respiratory tract infections					
Randomized trial vitamin D supplementation to prevent seasonal influenza A in schoolchildren. Influenza children.	Urashima et al., 2010 [156]	Japan	Over 300 schoolchildren	1200 IU/die during winter months	Reduced influenza A infections.
Effect on the incidence of pneumonia of vitamin D supplementation by quarterly bolus dose to infants in Kabul: a randomised controlled superiority trial.	Manaseki-Holland et al., 2012 [158]	Afghanistan	Over 3000 children	100,000 IU once every 3 months for 18 months	No decrease in incidence of pneumonia.
Randomized trial of vitamin D supplementation and risk of acute respiratory infection in Mongolia.	Camargo et al., 2012 [159]	Mongolia	247 children	Milk fortified with vitamin D from January to March	Significantly lower ARI episodes during the study period. Baseline serum vitamin D level: 7 ng/mL.
Effects of vitamin D supplements on influenza A illness during the 2009 H1N1 pandemic: a randomized controlled trial.	Urashima et al., 2014 [160]	Japan	247 high school students	2000 IU/day for 2 months	No decrease in incidence of influenza A infections.
Reduced primary care respiratory infection visits following pregnancy and infancy vitamin D supplementation: a randomised controlled trial.	Grant et al., 2015 [163]	New Zealand	Healthy pregnant women and their infants up to 6 months of age	Standard daily dose (1000 IU/400 IU) vs. high dose (2000 IU/800 IU)	Less primary care visits for ARI up to age 18 months.
Preventive effects of vitamin D on seasonal influenza A in infants: a multicenter, randomized, open, controlled clinical trial.	Zhou et al., 2018 [167]	China	400 infants	Low dose (400 IU) vs. high dose (1200 IU) daily for 4 months	More frequent influenza A infection in the low dose group.
Vitamin D Supplementation Associated with Acute Respiratory Infection in Exclusively Breastfed Infants.	Miao Hong et al., 2019 [168]	China	Infants up to 6 months	400–600 IU/day from birth to 6 months of age	Longer period before the first ARI episode in infants with supplementation.
Effect of Vitamin D supplementation to reduce respiratory infections in children and adolescents in Vietnam: A randomized controlled trial.	Loeb et al., 2019 [170]	Vietnam	1330 healthy children and adolescents	14,000 IU/week for 8 months	Similar incidence of influenza but moderately reduced incidence of other respiratory viral infections.

ARI, acute respiratory infection. PICU, pediatric intensive care unit.

**Table 7:** Main studies on the link between the omega-3 fatty acids supplementation and respiratory infections/illness in pediatric age.

Study	Author	Study Population	Omega-3 Fatty Acids (dosage)	Results
Infants fed docosahexaenoic acid- and arachidonic acid-supplemented formula have decreased incidence of bronchiolitis/bronchitis the first year of life.	Pastor et al., 2006 [214]	Infants RCT	DHA (17 mg/100 kcal)	Reduced incidence of bronchiolitis/bronchitis in the DHA+ group at 5 months (*p* = 0.0001), 7 months (*p* = 0.01), and 9 months (*p* = 0.01)
Fish oil n-3 polyunsaturated fatty acids selectively affect plasma cytokines and decrease illness in Thai schoolchildren: a randomized, double-blind, placebo-controlled intervention trial.	Thienprasert et al., 2009 [221]	Children (9–12 years) RCT	EPA (200 mg) + DHA (1 g)	Fewer episodes (*p* = 0.014) and shorter duration (*p* = 0.024) of illness in fish oil group. TGF-β1 concentration was lower in fish oil group (*p* < 0.001).
Enteral Omega-3 Fatty Acid, γ-Linolenic Acid, and Antioxidant Supplementation in Acute Lung Injury.	Rice et al., 2011 [222]	Adults within 48 h of ARI onset RCT	EPA (6.84 g) + DHA (3.40 g) daily	Not improving in the primary end point of ventilator-free days in patients with acute lung injury.
Prenatal Docosahexaenoic Acid Supplementation and Infant Morbidity: Randomized Controlled Trial.	Imhoff-Kunsch et al., 2011 [213]	Pregnant woman; a total of more of 800 infants were included in the trial. RCT	DHA (400 mg)	At 1 month: shorter duration of cough, phlegm, and wheezing, respectively (*p* < 0.001). At 3 months: 14% less time ill (*p* < 0.0001). At 6 months: shorter duration of fever, nasal secretion, difficulty breathing, rash, and “other illness,” respectively (all *p* < 0.05).
A Phase II Randomized Placebo-Controlled Trial of Omega-3 Fatty Acids for the Treatment of Acute Lung Injury.	Stapleton et al., 2011 [223]	Adults within 48 h of ARI onset RCT	EPA (9.75 g) + DHA (6.75 g) daily	Not reduction of biomarkers of pulmonary or systemic inflammation in patients with ALI.
Respiratory hospitalisation of infants supplemented with docosahexaenoic acid as preterm neonates.	Atwell et al., 2012 [215]	Infants born <33 weeks’ gestation RCT	High DHA (∼1%) vs. standard DHA (∼0.3%) in breast milk or formula	Not reduced hospitalisation for LRTI problems in the first 18 months.
The effect of a 1-year multiple micronutrient or n-3 fatty acid fortified food intervention on morbidity in Indian school children.	Thomas et al., 2012 [217]	Children (6–10 years) RCT	α-linolenic acid (900 mg) + DHA (100 mg) vs. α-linolenic acid (140 mg)	Significantly fewer episodes of URTI/child/year (relative risk (RR) = 0.88, 95% confidence interval (CI): 0.79, 0.97) in high consuming n-3 fatty acids group. Significantly shorter duration/episode of URTI (RR = 0.81, 95% CI: 0.78, 0.85), LRTI (RR = 0.91, 95% CI: 0.85, 0.97) in high consuming n-3 fatty acids group.
Effects of Growing-Up Milk Supplemented with Prebiotics and LCPUFAs on Infections in Young Children.	Chatchatee et al., 2014 [218]	Children (11–29 months) RCT	Growing-up milk with addition of 19.2 mg/100 mL of n-3 LCPUFAs (EPA + DHA, 4:6)	Decreased risk of developing at least 1 infection (*p* = 0.03) in the active group. Trend toward a reduction (*p* = 0.07) in the total number of infections in the active group.
N–3 Long-chain PUFAs reduce respiratory morbidity caused by iron supplementation in iron-deficient South African schoolchildren: a randomized, double-blind, placebo-controlled intervention	Malan et al., 2015 [219]	Children (6–11 years) with iron-deficiency RCT	EPA (80 mg) + DHA (420) + placebo vs Fe + EPA/DHA vs Fe + placebo vs placebo + placebo	Iron supplementation increased morbidity (*p* = 0.001), mostly respiratory. Increase in morbidity caused by iron supplementation was prevented (*p* = 0.009) by DHA/EPA.
Fish Oil–Derived Fatty Acids in Pregnancy and Wheeze and Asthma in Offspring	Bisgaard et al., 2016 [216]	Pregnant women at 24 weeks of gestation; a total of 695 children were included in the trial. RCT	2.4 g of n−3 LCPUFA (55% EPA and 37% DHA) daily	Lower risk of persistent wheeze or asthma in the treatment group (*p* = 0.035). Reduced risk of infections of the lower respiratory tract (*p* = 0.033) in the treatment group

ARI, acute respiratory infection; CI, confidence interval; DHA, docosahexaenoic acid; EPA, eicosapentaenoic acid; LCPUFA, long-chain polyunsaturated fatty acid; LRTI, lower respiratory tract infection; PUFA, polyunsaturated fatty acid; RCT, randomized controlled trial; RR, relative risk; TGF, transforming growth factor; URTI, upper respiratory tract infection.

**Table 8:** Efficacy of zinc in treating respiratory illnesses in childhood.

Study	Author	Country	Study Population	Dosage	Results
Randomized, double-blind, placebo-controlled clinical trial of the efficacy of treatment with zinc or vitamin a in infants and young children with severe acute lower respiratory infection.	Mahalanabis et al., 2004 [253]	India	153 children hospitalized with severe ALRI	10 mg twice daily for 5 days	Significantly reduced duration of fever and very ill status in boys, but not in girls.
Efficacy of zinc in the treatment of severe pneumonia in hospitalized children <2 y old.	Bose et al., 2006 [254]	India	299 children hospitalized with severe pneumonia	20 mg/day during hospital stay	No overall effect on the duration of hospitalization or of clinical signs associated with severe infection.
A randomized controlled trial of the effect of zinc as adjuvant therapy in children 2–35 months of age with severe or nonsevere pneumonia in Bhaktapur, Nepal.	Valentiner-Branth et al., 2010 [255]	Nepal	Over 2000 children with pneumonia	10 mg if aged 2–11 months, 20 mg if aged > or =12 months, for 14 days	No decrease in risk of treatment failure nor accelerated recovery.
A randomized controlled trial of oral zinc in acute pneumonia in children aged between 2 months and 5 years.	Ganguly et al., 2011 [256]	India	98 children with pneumonia	10 mg/day if <1 year, 20 mg if >1 year of age	No improvement in symptom duration.
The efficacy of zinc supplementation on outcome of children with severe pneumonia. A randomized double-blind placebo-controlled clinical trial.	Valavi et al., 2011 [257]	Iran	128 children with severe pneumonia	2 mg/kg/day, max 20 mg, for 5 days	Shorter time for symptoms resolution and shorter hospital stay.
Zinc Supplementation in Severe Acute Lower Respiratory Tract Infection in Children: A Triple-Blind Randomized Placebo Controlled Trial.	Bansal et al., 2011 [258]	India	Children hospitalized with ALRI	20 mg/day for 5 days	No decrease in recovery time or in duration of hospital stay.
Role of zinc in severe pneumonia: a randomized double bind placebo-controlled study.	Shah et al., 2012 [259]	Nepal	Children hospitalized for severe pneumonia	20 mg/die for 7 days	No effect on clinical recovery from severe pneumonia.
A randomized controlled trial of zinc as adjuvant therapy for severe pneumonia in young children.	Basnet et al., 2012 [260]	Nepal	610 children with severe pneumonia	10 mg if <11 months, 20 mg/day if >11 months, for 14 days	Slightly faster recovery, slightly lower risk of treatment failure but not statistically significant.
Zinc adjunct therapy reduces case fatality in severe childhood pneumonia: a randomized double-blind placebo-controlled trial.	Srinivasan et al., 2012 [261]	Uganda	352 children with severe pneumonia	20 mg if ≥12 months, 10 mg if <12 months, for 7 days	No significant effect on time to resolution of symptoms; decreased mortality rate.
Efficacy of zinc given as an adjunct in the treatment of severe and very severe pneumonia in hospitalized children 2–24 months of age: a randomized, double-blind, placebo-controlled trial.	Wadhwa et al., 2013 [262]	India	550 children hospitalized with severe pneumonia	20 mg/day during hospital stay	No difference in time to recovery.
Zinc as an adjunct to the treatment of severe pneumonia in Ecuadorian children: a randomized controlled trial.	Sempertegui et al., 2014 [263]	Ecuador	450 children hospitalized with pneumonia	20 mg/day during hospital stay	No effect on time to pneumonia resolution nor in treatment failure rate. Higher basal zinc concentrations associated with faster resolution of chest indrawings.
Effect of zinc supplementation on infants with severe pneumonia.	Yuan et al., 2016 [264]	China	73 infants hospitalized for pneumonia	10 mg/day if <6 mo; 20 mg/day if >6 mo during hospital stay	No improvements on clinical outcomes.
Zinc as an adjunct therapy in the management of severe pneumonia among Gambian children: randomized controlled trial.	Howie et al., 2018 [265]	Gambia	Over 600 children with severe pneumonia	10 mg if <12 mo; 20 mg if > 20 mo, for 7 days	No benefit in treatment failure rates or time to recovery from respiratory symptoms; marginal benefit in rapidity of resolution of chest indrawings.
Zinc supplementation promotes a th1 response and improves clinical symptoms in fewer hours in children with pneumonia younger than 5 years old. A randomized controlled clinical trial.	Acevedo-Murillo et al., 2019 [266]	Mexico	103 children hospitalized for pneumonia	10 mg/day if <1 year; 20 mg/day if >1 year during hospital stay	Quicker improvement in clinical status, oxygen saturation and respiratory rate. Patients’ baseline zinc levels were below normal range.
A randomized controlled trial of zinc supplementation in the treatment of acute respiratory tract infection in Thai children.	Rerksuppaphol et al., 2019 [267]	Thailand	64 hospitalized children with ALRI	30 mg/day during hospital stay	Faster symptoms resolution and shorter hospital stay.

ALRI, acute lower respiratory tract infection.

**Table 9:** Efficacy of zinc in preventing respiratory illnesses in childhood.

Study	Author	Country	Study Population	Dosage	Results
Effect of zinc supplementation between 1 and 6 months of life on growth and morbidity of Bangladeshi infants in urban slums.	Osendarp et al., 2002 [269]	Bangladesh	300 infants from Dhaka slum area, from 4 weeks to 24 weeks of age	5 mg/day for 20 weeks	Reduced incidence of ALRI and greater weight gains.
Effect of zinc supplementation started during diarrhea on morbidity and mortality in Bangladeshi children: community randomised trial.	Baqui et al., 2002 [270]	Bangladesh	8070 children	20 mg/day for 14 days in case of diarrhea episodes	Reduced incidence of diarrhea and ALRI.
Simultaneous weekly supplementation of iron and zinc is associated with lower morbidity due to diarrhea and acute lower respiratory infection in Bangladeshi infants.	Baqui et al., 2003 [271]	Bangladesh	799 infants	Different micronutrient formulations, some including zinc 20 mg weekly for 6 months	Lower risk of severe ALRI in the group receiving iron + zinc.
Effect of weekly zinc supplements on incidence of pneumonia and diarrhea in children younger than 2 years in an urban, low-income population in Bangladesh: randomised controlled trial.	Brooks et al., 2005 [272]	Bangladesh	1621 children aged 60 days to 12 months	70 mg weekly for 12 months	Reduced pneumonia incidence and mortality.
The prophylactic and therapeutic effectiveness of zinc sulphate on common cold in children.	Kurugol et al., 2006 [273]	Turkey	200 children	15 mg/day for 7 months, increased to 30 mg/day during colds	Significantly lower mean number of colds and duration of symptoms.
Zinc and iron supplementation and malaria, diarrhea, and respiratory infections in children in the Peruvian Amazon.	Richard et al., 2006 [274]	Perù	855 children	Iron, zinc (20 mg/day), or iron + zinc for 7 months	No statistically significant effect on incidence of respiratory infections.
A double-blind, randomized, clinical trial of the effect of vitamin A and zinc supplementation on diarrhea and respiratory tract infections in children in Mexico City, Mexico.	Long et al., 2006 [275]	Mexico	736 children living in a peri-urban area	Vitamin A vs. 20 mg zinc daily vs. vitamin A + zinc vs. placebo for 12 months	No effect on the incidence of respiratory infections.
Zinc or multiple micronutrient supplementation to reduce diarrhea and respiratory disease in South African children: a randomized controlled trial.	Luabeya et al., 2007 [276]	South Africa	Over 370 children from a rural community	Vitamin A vs. zinc 10 mg/day + vitamin A vs. a multi micronutrients supplement, from 6 to 24 months of age	No reduction in diarrhea and respiratory morbidity.
Effect of zinc supplementation on mortality in children aged 1–48 months: a community-based randomised placebo-controlled trial.	Sazawal et al., 2007 [277]	Zanzibar	42,546 children	10 mg/day (5 mg in children younger than 12 months) until 48 months of age	Non-significant reduction in the relative risk of all-cause mortality.
Adding zinc to supplemental iron and folic acid does not affect mortality and severe morbidity in young children.	Bhandari et al., 2007 [278]	India	94,359 children	10 mg daily for 12 months	No significant difference in death and hospitalization rates.
Effect of daily zinc supplementation on child mortality in southern nepal: a community-based, cluster randomised, placebo-controlled trial.	Tielsch et al., 2007 [279]	Nepal	Over 40,000 children	zinc 10 mg/day vs. other supplements vs. placebo for 12 months	Not significantly different mortality rate sand frequency of respiratory infections.
Zinc supplementation for prevention of acute respiratory infections in infants: a randomized controlled trial.	Malik et al., 2014 [280]	India	272 children with acute respiratory infections	20 mg/day for 2 weeks	Decrease in duration of the episode, lower frequency of future ALRIs.
Occurrence of infections in schoolchildren subsequent to supplementation with vitamin D-calcium or zinc: a randomized, double-blind, placebo-controlled trial.	Mandlink et al., 2020 [281]	India	435 schoolchildren	(Children in the zinc arm)10 mg/day for 7 months	No significant reduction in the occurrence of infections.

ALRI, acute lower respiratory tract infection.
